# Supplementary material for: Genomic adaptation of an autochthonous cider yeast strain to buckwheat and barley wort under stressful brewing conditions
Source: Appl Environ Microbiol. 2025 Oct 8;91(11):e01015-25. doi: 10.1128/aem.01015-25 (PMC12628810; doi:10.1128/aem.01015-25)
Supplement: Supplemental figures — Figures S1 to S9. [file aem.01015-25-s0001.pdf]

Supplementay Figures

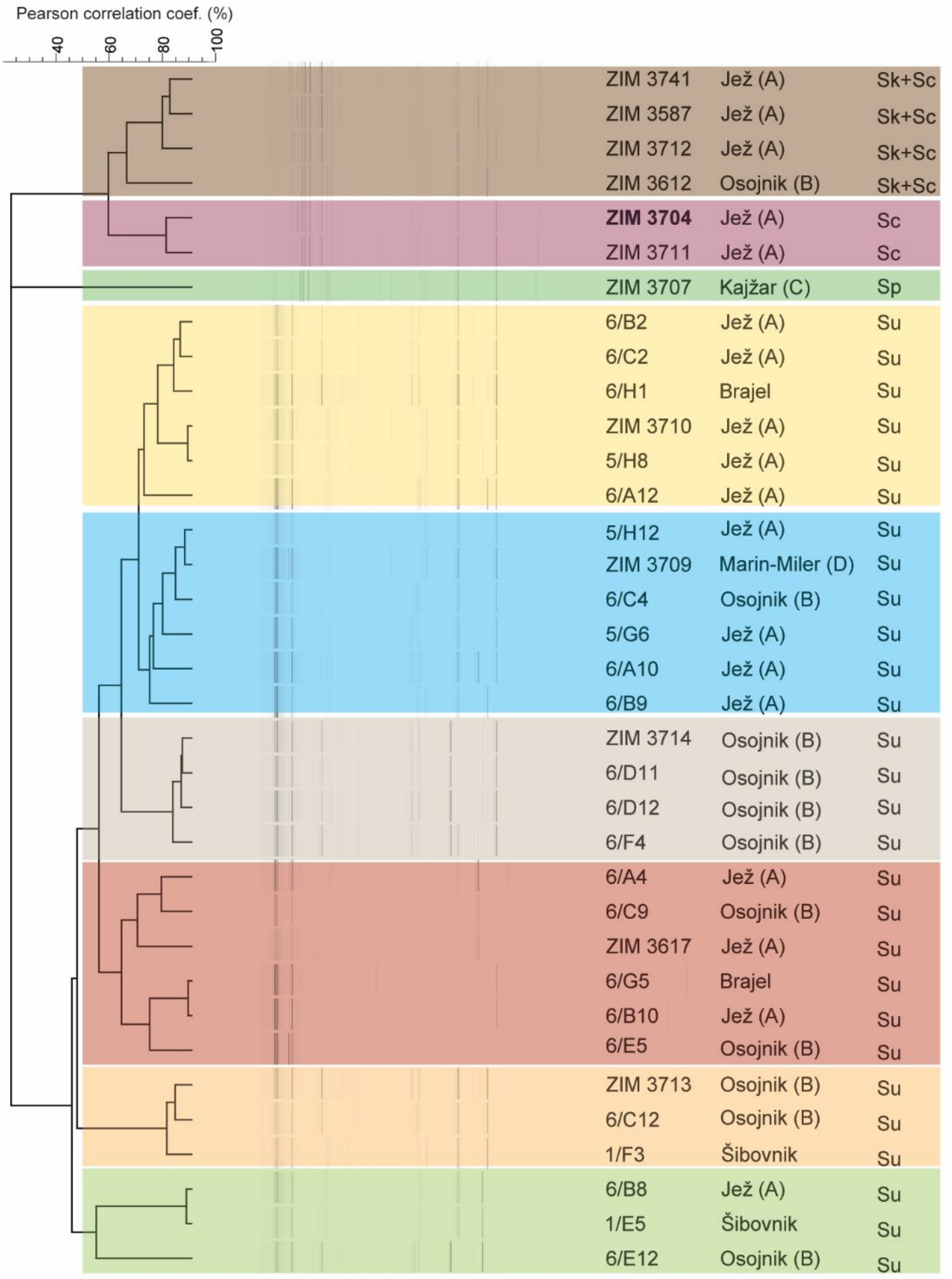

Figure S1. Dendrogram of PCR fingerprints of *Saccharomyce*s spp. isolates from cider based on seven microsatellite loci.

Seven microsatellite regions (SuARS409, SuYBR049C, SuYKR045C, SuYHR042-043, SuHTZ1PLB3, SuYHR102W, SuYIL130W) of strains listed in Suppl. Table 1 were amplified by PCR using primers described by Masneuf-Pomarede *et al.* (13). The representative isolates designated with acronym of Collection of Industrial Microorganisms (ZIM) were tested for their brewing characteristics. Two isolates, 5/G6 and 5/H12 were not genotyped.

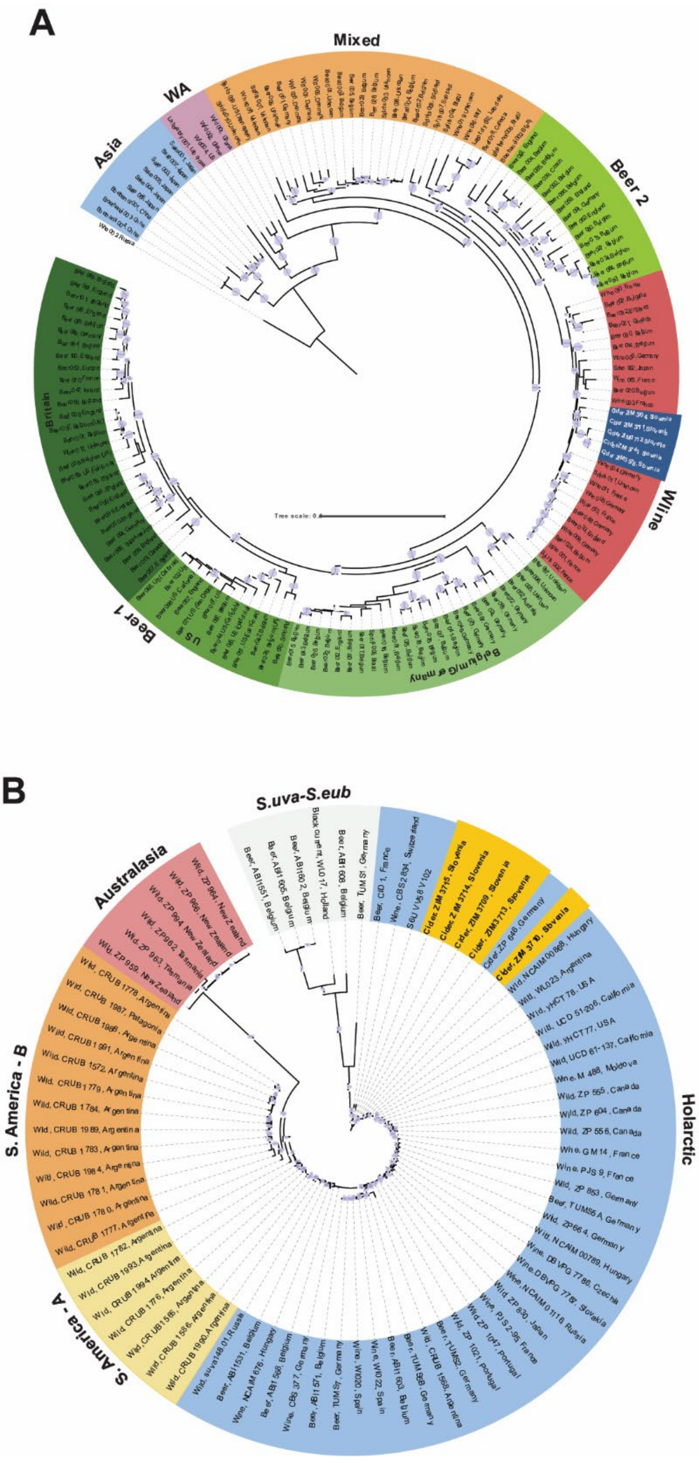

Figure S2. Phylogenetic placement of autochtoneous cider strains of *Saccharomyces*

A. Phylogenetic placement *of S. cerevisiae* cider strains in the wine clade of industrial *S. cerevisiae* yeasts from Gallone et al. (10). The phylogeny was inferred from 147 sequences and 134686 SNPs using the Maximum Likelihood method as implemented in IQ-TREE with the TVM+F+R5 model of sequence evolution. Branch lengths correspond to the expected number of substitutions per site. Cider S.cer strains are colored in blue.

B. Phylogenetic placement of *S. uvarum* cider strains in the Holarctic clade of *S. uvarum* populations from Almeida et al. (17) and Gallone et al. (50). The phylogenetic tree was constructed based on 55 sequences and 1333656 SNPs using the Maximum Likelihood method as implemented in IQ-TREE with the TVM+F+R4 model. Cider S.uva strains are colored in yellow.

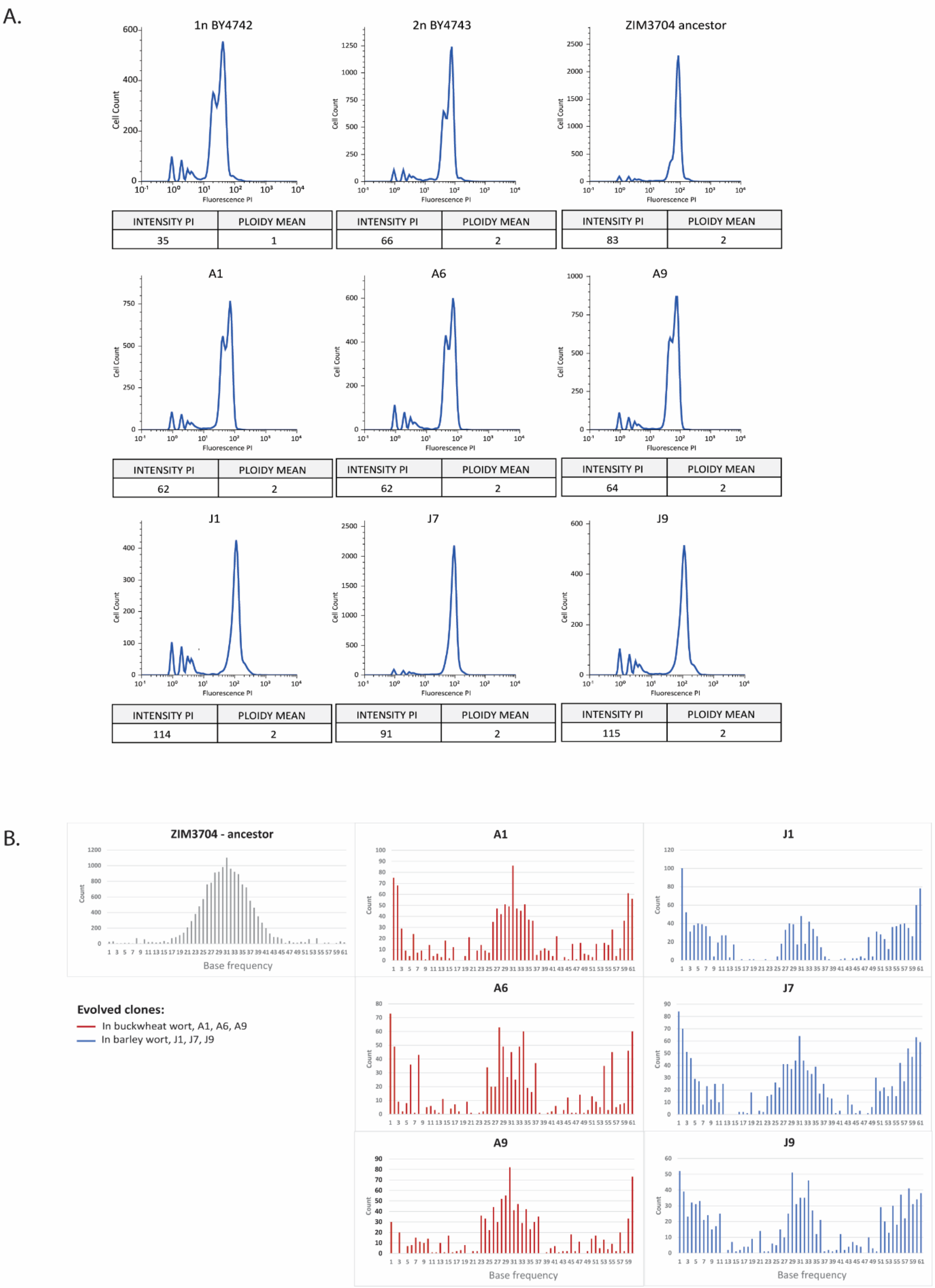

Figure S3. Ploidy determination of the autochtoneous cider strain *S. cerevisiae* ZIM 3704 (ancestor) and its evolved clones on buckwheat (A1, A6, A9) and barley (J1, J7, J9) worts after 30 fermentations bottelnecks.

- **A.** Flow cytometry profiles, intensity of propidium iodide-stained cells, and predicted ploidy from control laboratory strains were used to determine the ploidy of ancestor and evolved clones in buckwheat and barley worts.
- **B.** Allelic frequency patterns found in ancestor and evolved clones using nQuire.

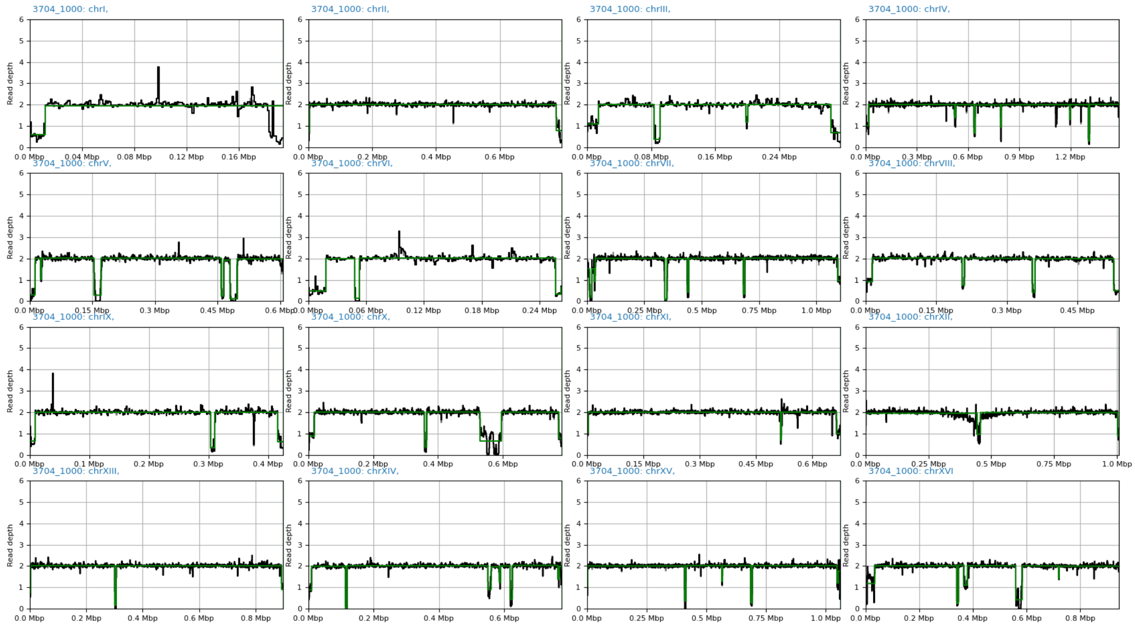

Figure S4. Depth of coverage across 16 chromosomes of the ancestral strain ZIM 3704 from cider, shown in 100-kb windows. Plots were generated using CNVpytor (69).

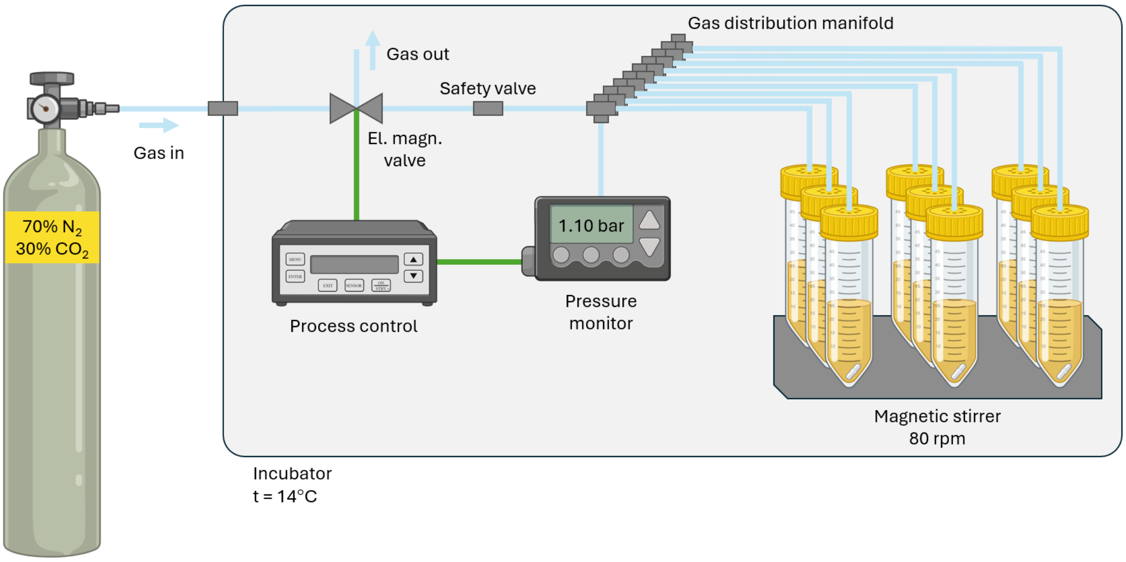

Figure S5. A high-pressure control system integrated into the fermenters to simulate the combined hydrostatic and dissolved CO_2 pressures representative of the midpoint in 20-meter-high industrial conical fermenters used for lager beer production.

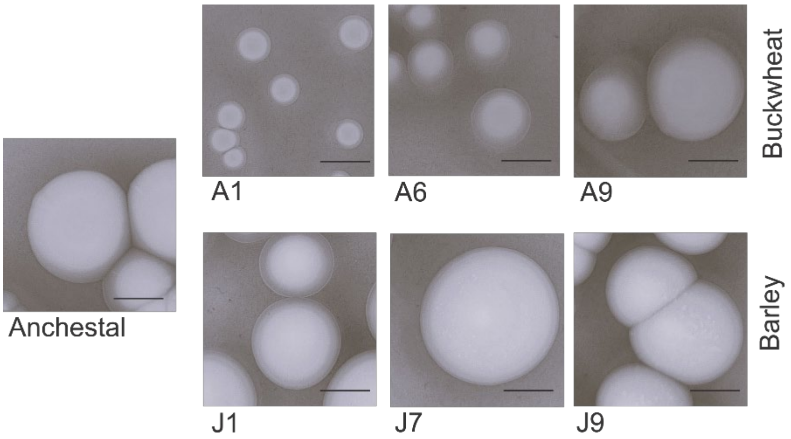

Figure S6. Morphologically distinct clones isolated from biomass following adaptive laboratory evolution of 30 bottleneck fermentations, each lasting 7 days in buckwheat and barley worts under stressful conditions. Scale bar, 0.5 cm.

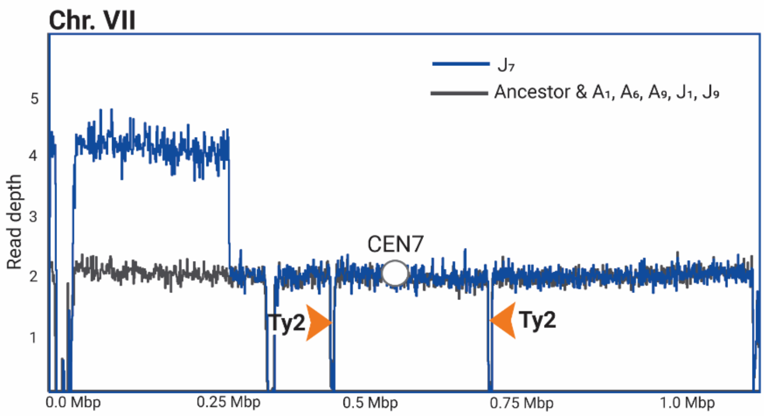

Figure S7. Large segmental duplication on chromosome VII in the evolved clone J_7 (blue line) compared to the ancestor and evolved clones A_1, A_6, A_9, J_1, and J_9 (grey lines). The duplicated region is located 160 kb away from a leftward, mirror-oriented Ty2 element pair (orange arrows). CEN7, centromere of chromosome VII.

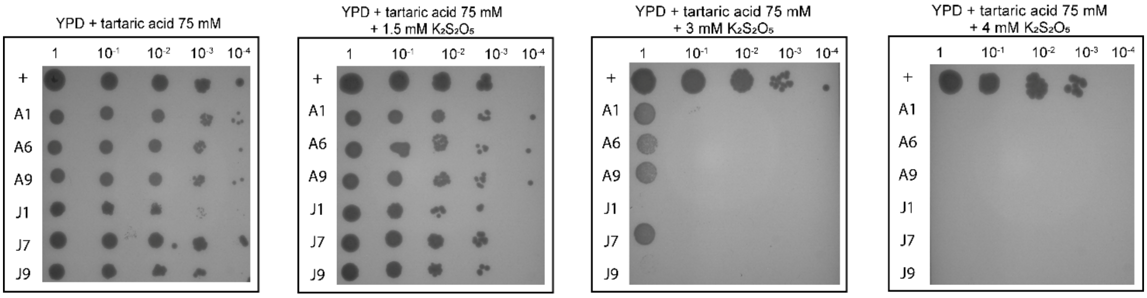

Figure S8. Spotting assay for testing the change in sulfite tolerance of evolved clones (A_1, A_6, A_9, J_1, J_7, J_9) in comparison to ancestor strain (+).

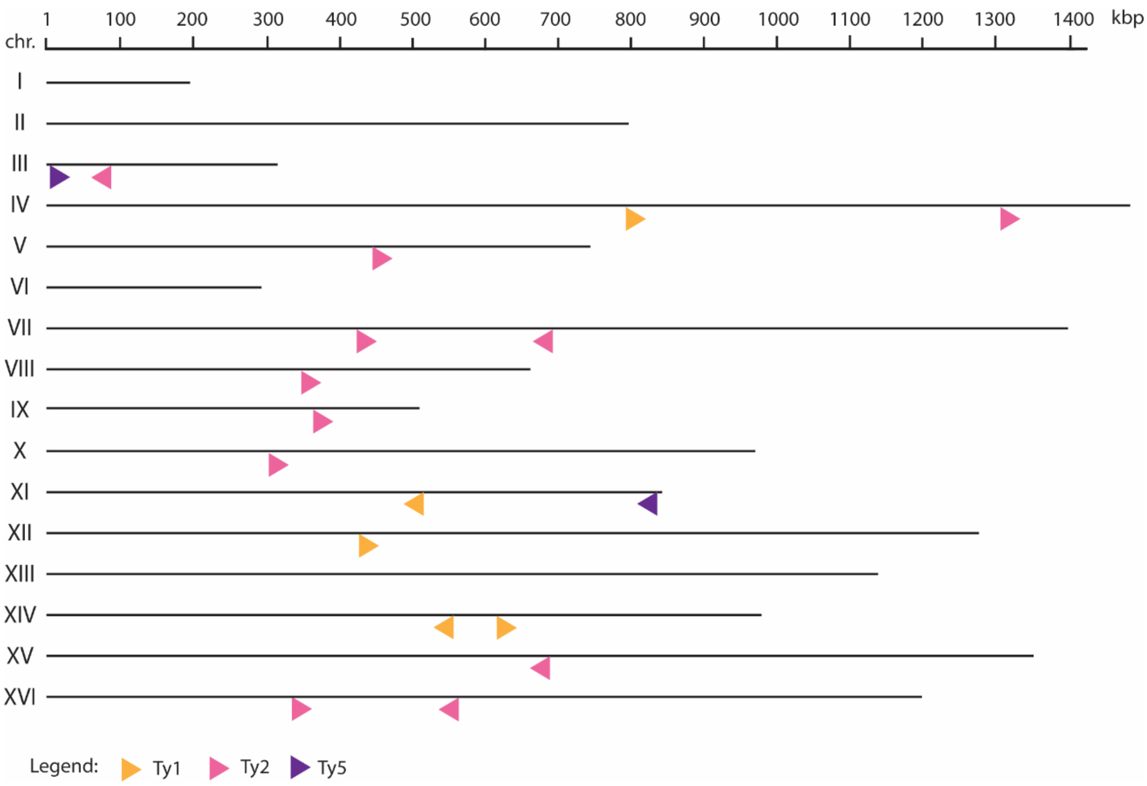

Figure S9. Ty elements of Ty1, T2 and Ty5 of the ancestor strain.
